# The epidemiology and impact of persistent *Campylobacter* infections on childhood growth among children 0–24 months of age in resource-limited settings

**DOI:** 10.1016/j.eclinm.2024.102841

**Published:** 2024-09-28

**Authors:** Francesca Schiaffino, Josh M. Colston, Maribel Paredes Olortegui, Pablo Peñataro Yori, Evangelos Mourkas, Ben Pascoe, Aldo A.M. Lima, Carl J. Mason, Tahmeed Ahmed, Gagandeep Kang, Estomih Mduma, Amidou Samie, Anita Zaidi, Jie Liu, Kerry K. Cooper, Eric R. Houpt, Craig T. Parker, Gwenyth O. Lee, Margaret N. Kosek

**Affiliations:** a Faculty of Veterinary Medicine, Universidad Peruana Cayetano Heredia, San Martin de Porres, Lima, Peru; b Division of Infectious Diseases and International Health, School of Medicine, University of Virginia, Charlottesville, VA, USA; c Asociacion Benefica Prisma, Iquitos, Peru; d Ineos Oxford Institute for Antimicrobial Research, Department of Biology, University of Oxford, Oxford, United Kingdom; e Centre for Genomic Pathogen Surveillance, Big Data Institute, University of Oxford, Oxford, United Kingdom; f Institute of Biomedicine for Brazilian Semiarid, Faculty of Medicine, Federal University of Ceará, Fortaleza, Brazil; g Armed Forces Research Institute of Medical Sciences, Bangkok, Thailand; h Nutrition and Clinical Services Division, International Centre for Diarrhoeal Disease Research, Bangladesh (icddr,b), Dhaka, Bangladesh; i Wellcome Research Unit, Christian Medical College, Vellore, India; j Haydom Lutheran Hospital, Manraya, Tanzania; k University of Venda, Limpopo Province, South Africa; l Division of Women and Child Health, Aga Khan University, Karachi, Pakistan; m School of Public Health, Qingdao University, Qingdao, China; n School of Animal and Comparative Biomedical Sciences, University of Arizona, Tucson, AZ, USA; o Agricultural Research Service, U.S. Department of Agriculture, Produce Safety and Microbiology Research Unit, Albany, CA, USA; p Rutgers Global Health Institute & Department of Biostatistics and Epidemiology, School of Public Health, Rutgers the State University of New Jersey, Newark, NJ, USA

**Keywords:** Persistent infections, Campylobacteriosis, Carriage, MAL-ED

## Abstract

**Background:**

*Campylobacter* is the leading cause of bacterial gastroenteritis worldwide. It is generally associated with an acute gastrointestinal infection causing a self-limiting diarrheal episode. However, there is evidence that persistent/recurrent carriage of *Campylobacter* also occurs. In hyperendemic settings the epidemiology and consequences of persistent *Campylobacter* enteric infections is poorly studied.

**Methods:**

Risk factors for and growth consequences of persistent *Campylobacter* infections detected by polymerase chain reaction (qPCR) were evaluated with data from the MAL-ED birth cohort study in children 0–24 months of age between November 2009 and February 2012. A persistent *Campylobacter* infection was defined as three or more consecutive *Campylobacter* positive monthly stools.

**Findings:**

Across all study sites, 45.5% (781/1715) of children experienced at least one persistent *Campylobacter* episode. The average cumulative duration of days in which children with persistent *Campylobacter* were positive for *Campylobacter* spp. was 150 days (inter-quartile range: 28–236 days). Children who experienced a persistent *Campylobacter* episode had an attained 24-month length-for-age (LAZ) score that was 0.23 (95% (CI): −0.31, −0.15) less than children without a persistent *Campylobacter* episode. Among children who had at least one episode of *Campylobacter* over a 3-month or 9-month window, persistent episodes were not significantly associated with poorer 3-month weight gain (−28.7 g, 95% CI: −63.4 g, 6.0 g) but were associated with poorer 9-month linear growth (−0.134 cm 95% CI: −0.246, −0.022) compared to children with an episode that resolved within 31 days.

**Interpretation:**

Persistent/recurrent *Campylobacter* infection is common among children and has a measurable negative impact on linear growth in early childhood.

**Funding:**

Funding for this study was provided by the 10.13039/100000865Bill and Melinda Gates Foundation (OPP1066146 and OPP1152146), the 10.13039/100000002National Institutes of Health United States (R01AI158576 and R21AI163801 to MNK and CTP; K43TW012298 to FS; K01AI168493 to JMC; GOL was supported by K01AI145080. This research was also supported in part by USDA-ARS CRIS project 2030-42000-055-00D. The funders had no role in study design, study implementation, data analysis, or interpretation of the results.

**Research in context**

**Evidence before this study**

Persistent infections by *Campylobacter* infection have been previously documented, yet they remain an understudied entity in the field. On January 15, 2024, we searched of PubMed for studies using the following search terms: “*Campylobacter*” AND (“carriage” OR “persistent infection” OR “recurrent infection”), without language restrictions. We reviewed 260 articles and selected research studies that described *Campylobacter* infections that were persistent or recurrent in nature, irrespective of the definition utilized by the authors. *Campylobacter* was described in both high- and low-income countries with evidence of length of over a month, but studies were descriptive in nature and limited to studies in which results of <100 infections were characterized. *Campylobacter* carriage and persistence had been preliminary described in the MAL-ED cohort study by Amour et al. 2016 (28.9% of children had a persistent *Campylobacter* infection during the first year of life). However, the data set utilized for this analysis consisted of ELISA in which testing was restricted to once every 3 months in the second year of life. However, the risk factors and consequences of persistent infections have not been studied systematically.

**Added value of this study**

This study from the MAL-ED cohort study by including monthly qPCR for the first and second year of life, as well as all diarrheal episodes. This provided us with the adequate data set to define persistent infections appropriately, increasing the availability and temporal resolution of test results. In the second year of life in this cohort, for an initial analysis 11,270 ELISA results are available, while for this study 21,288 qPCR results are available. Standardized sampling across the lifespan of these children allowed for improved and homogeneous case definitions. This data set provided additional resolution of the timing of the infection which allowed for modelling of the effects of *Campylobacter* infections (persistent and not persistent) on growth over shorter periods of time to allow for the temporality of association to be assessed and to determine if duration of the episode has a dose type effect on ponderal and linear growth. This study provides strong evidence that persistent *Campylobacter* infections are a common phenomenon in early childhood, and that persistent *Campylobacter* infections negatively impact childhood growth.

**Implications of all the available evidence**

The treatment of *Campylobacter* in non-immunocompromised hosts has historically recommended treatment only in cases in pregnancy and in cases with high fevers, dysentery, and prolonged symptomatic gastroenteritis of >1 weeks’ duration. These findings call into question this routine set of guidelines as many of these infections are only mildly symptomatic, but have adverse sustained impact on child growth and attained height at 24 months of age.

## Introduction

In the early decades of the 20th century, Mary Mallon, known more infamously as ‘Typhoid’ Mary, was shown to have a persistent gastrointestinal infection two years during her initial confinement.^1^ Despite this early evidence of persistent gastrointestinal infection, most modern efforts to understand gastrointestinal pathogens focus on acute infection. However, several enteric pathogens have shown a propensity for persistent infection in addition to the typhoid-fever causing *Salmonella*,^2^ *Helicobacter pylori,*^3^ and *Cryptosporidium.**^4^* These persistent infections are often associated with long-term adverse consequences for child health and development including chronic intestinal inflammation, growth faltering and gastrointestinal cancer.^5^, ^6^, ^7^, ^8^, ^9^, ^10^

*Campylobacter* is the most prevalent bacterial causes of bacterial gastroenteritis in both high and low- and middle-income countries.^11^, ^12^, ^13^, ^14^, ^15^ Persistent or recurrent infections with this pathogen have been documented.^16^ The duration of *Campylobacter* carriage following a diarrhea episode is highly variable with median infection lengths of 7–31 days,^17^, ^18^, ^19^, ^20^ however what is consistent across the literature is that a substantial fraction of the infections lasts for over a month. In Thailand, 16% of infections exceeded a month,^17^ and in Canada, 33% exceeded a month.^19^ Although the health impacts of acute campylobacteriosis are relatively well documented, the importance of persistent/recurrent *Campylobacter* infection is less well understood. Findings from multi-site cohort studies show that both symptomatic and asymptomatic acute *Campylobacter* infections are associated with intestinal inflammation,^15^^,^^21^^,^^22^ reduced weight gain and poorer linear growth, resulting in stunting in children living in resource poor areas of the world.^15^^,^^21^^,^^23^ It is logical to hypothesize that persistent infections may compound these negative developmental consequences. Additionally, persistent infection and relapsing disease caused by *Campylobacter* is well recognized in immunocompromised patients, particularly individuals living with HIV or suffering from hypogammaglobulinemia.^24^, ^25^, ^26^, ^27^, ^28^ Recently two separate controlled human infection trials of immunocompetent individuals done with *Campylobacter jejuni* CG8421 that included adequate and appropriate treatment following exposure have been completed. Relapsing infection with the trial strain was evidenced in 5 of the 14 exposed individuals^29^ in one trial and 2 out of 23 in a second trial, demonstrating that relapse is not limited to populations with immunocompromise.^30^^,^^31^

Persistent *Campylobacter* infections among the pediatric population remain understudied,^29^^,^^32^ partly because of the need for serial sampling, and because guidelines do not advise the treatment of asymptomatic infections. The study is additionally complicated by the evolution of *Campylobacter* diagnostics, with a transition from culture based to either immune-based (ELISA) or nucleic acid-based diagnostics, each of which has different sensitivity and specificity which may affect the recognition of prolonged infections. Evidence from the MAL-ED Peru^33^ site, located in Amazonian city of Iquitos, revealed a high burden of persistent *Campylobacter* infections among children living in that resource constrained settings, but it remains unclear to what extent this phenomenon is present in other settings where longitudinal surveillance and frequent sampling for *Campylobacter* infection takes place.^34^ To address this, this study aimed to describe the incidence of persistent *Campylobacter* infections, and to estimate the risk factors for and growth consequences of persistent *Campylobacter* infections in children 0–24 months of age enrolled in the MAL-ED cohort study.

## Methods

### Study population

The MAL-ED study was conducted in eight sentinel communities, each in different countries: Bangladesh (BGD), Brazil (BRA), India (IND), Nepal (NPL), Pakistan (PAK), Peru (PER), Tanzania (TAZ), and South Africa (ZAF). The sites were selected purposively based on a balance of considerations including known diarrheal disease endemicity, representativeness of both urban and rural settings as well as the demographic profile of the regions in which they are located, and each of the regions of Africa, South Asia and Latin America. The selection of eight sites from three continents in both urban and rural settings maximized both internal validity and generalizability of findings. The target sample size was calculated to give statistically significant estimates of the incidence of diarrheal disease (of all duration and etiology) in each of the study sites. The study protocol has been published previously as have the principal findings.^35^ Briefly, between November 2009 and February 2012, 2145 infants were enrolled within 17 days of birth, and 1715 completed two years of follow-up. Household visits were conducted twice per week to ascertain daily symptoms and illness generating a continuous registry of illness among the cohort. The study protocol has been published previously as have the principal findings.^36^, ^37^, ^38^, ^39^, ^40^

Ethical approval for MAL-ED was given by the respective Institutional Review Boards and regional health authorities. Details of each institutional review board are presented in the ethical declarations. Written informed consent was obtained from all participants’ caregivers.

### Stool collection and processing

Stool samples were collected at monthly intervals for surveillance purposes. Additionally, stool samples were collected every time a child experienced an episode of diarrhea. The microbiology methods used in the MAL-ED cohort study have been previously published.^41^ Two different *Campylobacter*-specific diagnostic methods from stool samples were included in the analysis: quantitative polymerase chain reaction (qPCR) and ELISA.

ELISA test results were available for the testing of monthly surveillance stool in the first year of life and quarterly surveillance stools in the second year of life and for the testing of stools from children with diarrhea (26,809 stools in the first year of life and 11,270 stools in the second year of life). Results for qPCR (performed later on the biorepository of extant samples), by contrast, were available for monthly surveillance stools in both the first and second year of life and in cases of diarrhea for children who completed 24 months of surveillance (totaling 20, 140 stools in the first year of life and 21, 288 stools in the second year of life). Details on both methodologies are briefly described here:

*Quantitative Polymerase Chain Reaction using TaqMan Array cards*^42^, ^43^, ^44^, ^45^: Total nucleic acids were extracted from 200 mg of stool using the QIAamp Fast DNA stool mini kit (Qiagen). Phocine herpesvirus and MS2 bacteriophage were utilized as extrinsic controls. A total of 20 μL of DNA were added to 50 μL of Ag-Path-ID One-Step RT-PCR buffer, 4 μL enzyme mix, and 26 μL of water for a total reaction volume of 100 μL. qPCR was done using TaqMan Array cards for the detection of 29 enteric pathogens.^43^^,^^44^ The cut-off used to determine pathogen positivity was 35 quantification cycles (Cq). Quantitative analysis of *Campylobacter* load was not done beyond detection as a binary variable was as analysis has not demonstrated that copy number is associated with disease status (diarrhea/non-diarrhea) or growth deficits.^42^^,^^46^ *Campylobacter* spp. was detected based on a 60 kDa chaperonin, *Cpn60*, sequence^47^ modified by Liu and collaborators.^44^ Primers and probes include: Cpn60_Fw1: AAAGTIGGMAAAGATGGTGTTAT, Cpn60_Fw2: AAAGTIGGWAAAGACGGYGTTAT, Cpn60_Rv1: TCAAATTGCATACCYTCAAC, Cpn60_Probe1: TTTGCCTCTTCMACAGT, Cpn60_Probe 2: TTTGCTTCTTCWACAGT designed to detect diverse *Campylobacter* species.

*Enzyme Linked Immunosorbent Assay:* The ProSpecT *Campylobacter* ELISA was utilized on fecal samples following manufacturer's instructions.^48^

Supplementary File 1 presents additional details on qPCR and ELISA methodology.

### Definition of persistent *Campylobacter* episodes

Persistent *Campylobacter* infections cannot be differentiated from recurrent reinfections with epidemiologic data alone. As a result, we will herein refer to both of alternatives as persistent carriage. Persistent carriage was defined according to the criteria previously proposed by Rouhani and colleagues^34^ as three or more consecutive *Campylobacter* spp. positive monthly stools (including when the monthly sample was diarrheal). Positive monthly samples separated by missing results, either because there was no diagnostic result, or because no monthly sample was collected, were counted as discrete (not persistent) episodes. To characterize the distribution of *Campylobacter* episode duration, episode length was defined as the number of days between an initial positive stool sample (of either type) and the first of two subsequent consecutive negative samples spanning ≥30 days.

### Statistical analysis and data visualization

The duration of each *Campylobacter* episode, the total number of *Campylobacter* persistent episodes, and the number of children who experienced a *Campylobacter* persistent episode were calculated for each site and separately for the TaqMan Array Cards and ELISA diagnostic modalities. The distribution of the duration of *Campylobacter* episodes was visualized using violin plots to display the variability at different study sites. Incidence rates and 95% confidence intervals of *Campylobacter* persistence episodes over a follow-up period of 24 months were calculated for each site and for TaqMan Array Cards and ELISA, separately. Length for age (LAZ) and weight for age (WAZ) Z-scores were calculated based on the length and weight measurements obtained for each child using the WHO Child Growth Standards STATA *igrowup* package.^49^ LAZ values from Pakistan were not included in the analysis because quality assurance procedures identified an unexplained bias in a subset of length measurements.^37^

Since only 32% of the 50,000 *Campylobacter* results occurred concurrently (by exact date) with anthropometric assessments, of which there were 47,000, each anthropometric variable (LAZ, WAZ) was in turn had values interpolated to the exact date of the *Campylobacter* results using predictions from linear mixed effects models fitted to the anthropometric Z-scores, with fixed effects for sex, and age on that date (with terms up to fourth order polynomial), and participant- and site-specific random effects, as described elsewhere.^50^ Because of the greater temporal consistency and resolution in sample testing, qPCR results for *Campylobacter* were used in modeling outcomes related to growth analysis, while ELISA was used only for descriptive analysis. Statistical significance was achieved with a p-value <0.05. Data processing, analysis and visualization were performed in Stata 18 software and R (v.2022.07.02).^51^^,^^52^

#### Risk factors associated with persistent *Campylobacter* episodes

First, **to evaluate the risk factors associated with persistent *Campylobacter* episodes**, the occurrence of a persistence episode was treated as a time-fixed binary outcome (comparing children who experienced at least one persistent episode during follow-up to the reference category of children who did not experience any) (Model 1). Multivariable logistic regression models were fitted to this outcome with covariates that included sociodemographic characteristics (sex, the WAMI composite index of socio-economic status,^53^ maternal education (no education, primary education completed, secondary education completed, higher education completed), anthropometric characteristics (birth weight (kg), baseline LAZ and WAZ scores, LAZ and WAZ scores at 24 months of age), indicators of enteropathy burden (cumulative number of diarrhea episodes at 24 months of age, cumulative number of non-*Campylobacter* bacterial, protozoal and viral pathogens at 24 months of age), baseline household characteristics (number of household members per room for sleeping, (<2, 2–3, 3–4, ≥4),^54^ type of water source (improved vs. unimproved),^55^ type of sanitation infrastructure (improved vs. unimproved),^55^ materials of walls, roofs and floors (improved vs. unimproved),^56^ poultry ownership and cattle ownership), and site. Missing values for baseline variables varied from 7.3% to 17.5% and were simultaneously imputed using multivariate normal regression (MVN) with an iterative Monte Carlo method with site as an additional predictor under the assumption that they were missing at random. Variables considered for inclusion in the outcome models were based on known associations with *Campylobacter* infection, and those considered in the growth models were selected based on known associations with early childhood growth, both selected based on the existing literature.

#### Anthropometric consequences of associated with *Campylobacter* persistent episodes

To **evaluate changes in LAZ scores at 24 months of age due to the persistent *Campylobacter* episodes, we performed two analyses**. First, the occurrence of a persistent *Campylobacter* episode was treated as the main exposure to evaluate their association with attained LAZ at 24 months of age, a durable indicator of child nutritional status. A multivariable linear regression model was fitted with attained 24-month LAZ as the time-fixed outcome, treating the occurrence of persistent episodes as a binary exposure variable (Model 2). The model was adjusted for similar sociodemographic, anthropometric, diagnostic, and household covariates, as well as fixed effects for site.

Second, to complement the analyses of attained length at 24 months of age, we modeled the association between *Campylobacter* infection and growth (change in weight and length) over shorter periods (i.e., during the period of infection) to associate *Campylobacter* temporally to acquired growth deficits. Growth over shorter time periods (weeks-to months) does not necessarily translate into long-term deficits in attained stature, because slower growth in one period can be followed by catch-up growth in a later period if further growth insults are not accrued.^57^ However, evidence that persistent *Campylobacter* is temporally associated with poorer interval growth strengthens causal inference of the association between *Campylobacter* and diminished attained length at 24 months of age.

For this second analysis, we constructed two sets of models. In the first, we aimed for comparability with our prior estimates of the impact of acute *Campylobacter* infection and child growth.^23^ We considered the same outcomes (change in weight over a 3-month period, and changes in length over a 9-month period) and main exposures (in the 3-month model, the presence/absence of symptomatic *Campylobacter* and asymptomatic *Campylobacter*, and in the 9-month model (Model 3), the total number of symptomatic and asymptomatic *Campylobacter* detections (Model 4)). All models included a random intercept for study site and a nested random intercept for each study child. Based on a visual inspection of the autocorrelation function as well as model fit, the covariance structure was fixed such that the residuals took a first-order autoregressive form for both change-in-weight and change-in-height models. We adjusted for site as a fixed effect, as well as socio-economic status (WAMI score), prior nutritional status (stunting and WHZ category, defined as WHZ less than −1, WHZ between −1 and 0, and WHZ greater than 0), sex, birthweight, episodes of diarrheal disease that were not associated *Campylobacter* in the same period, all of which is also similar to our prior work. We also adjusted for the presence of non-*Campylobacter* pathogens (any viral infection, any protozoal infection, and any non-*Campylobacter* bacterial infection) detected over the same period. Age terms were included as fractional polynomials, a method to adjust for curvi-linear relationships.^58^^,^^59^ These terms were defined separately for each model.

In the second set of models, we aimed to examine the association between acute episodes of *Campylobacter* and growth, vs persistent episodes of *Campylobacter* and growth. Differentiating this set of models from the first set, only intervals during which at least one episode of *Campylobacter* was detected, were included. The models compared changes in weight over 3-month intervals and length over 9-month intervals for children who experienced at least 1 prolonged but not non-persistent episode (32 days–89 days) during the interval and at least 1 persistent episode (90+ days), compared to those who experienced an acute episode that did not become persistent (<31 days). These models controlled for the same covariates as in the first set of models and did not use the interpolated anthropometric values.

A sensitivity analysis for all models was performed using the data set without multiple imputation of missing data. The output of this analysis did not change the main findings and conclusions of this study. The output is included in Supplementary Tables S1–S4.

### Ethical declarations

Institutional Review Boards and regional health authorities included: (BRA) The Institutional Review Board for Health Science Research of the University of Virginia, the Research Ethics Committee of Universidade Federal de Ceara, and the National Research Ethics Committee of the National Council of Health of Brazil; (BGD) The Institutional Review Board for Health Science Research of the University of Virginia and the Ethical Review Committee of ICDDR,B; (IND) The Institutional Review Board of the Christian Medical College of Vellore and the Health Ministry's Screening Committee of the Indian Council of Medical Research; (NPL) The Institutional Review Board of the Institute of Medicine of Bhaktapur, the Ethical Review Board of the Nepal Health Research Council, and the Institutional Review Board of the Walter Reed Army Institute of Research; (PER) The Institutional Review Board of the Johns Hopkins School of Public Health, the Ethics Committee of Asociacion Benefica PRISMA, and the Regional Health Department of Loreto; (PAK) The Ethical Review Committee of the Aga Khan University; (TAZ) The Institutional Review Board for Health Sciences Research of the University of Virginia and the Medical Research Coordinating Committee of the National Institute for Medical Research, and the Ministry of Health and Social Welfare of Tanzania; (ZAF) The Institutional Review Board for Health Science Research of the University of Virginia, the Department of Health and Social Development of Limpopo Province, and the Ethical Clearance Committee of the University of Venda.

### Role of the funding source

The funders had no role in study design, study implementation, data analysis, or interpretation of the results.

## Results

### Incidence and duration of persistent *Campylobacter* infection

Table 1 summarizes the burden of persistent *Campylobacter* infection detected in the study subjects by site and diagnostic modality. Tanzania and Bangladesh were the sites with the greatest number of persistent episodes according to the qPCR method, with 263 and 215 episodes, respectively. These were followed by Pakistan with 163 episodes, Nepal with 127 episodes, India with 109 episodes and Peru with 95 episodes. Taking ELISA as a diagnostic method, Pakistan and Bangladesh were the sites with the greatest number of persistent episodes (n = 264 and n = 224, respective), followed by Tanzania (n = 173), India (n = 137), Peru (n = 118) and Nepal (n = 96) (Table 1).

**Table 1:** Number, percent, and incidence rate of persistent *Campylobacter* spp. episodes among the eight MAL-ED sites.

Site	Average number of diarrhea samples analyzed per child	Average number of asymptomatic samples analyzed per child	Total number of *Campylobacter* persistence episodes		Percent of children who experienced a *Campylobacter* persistence episode		Incidence rates (95% CI)	
			qPCR	ELISA	qPCR^a^	ELISA	qPCR	ELISA
Bangladesh	6.34	21.33	215	224	69.0% (145/210)	66.8% (177/265)	0.47 (0.40–0.56)	0.74 (0.64–0.86)
Brazil	0.58	16.79	40	29	22.4% (37/165)	11.6% (27/232)	0.11 (0.08–0.15)	0.08 (0.05–0.11)
India	3.75	22.66	109	137	39.2% (89/227)	47.6% (117/246)	0.25 (0.20–0.31)	0.37 (0.31–0.44)
Nepal	4.56	24.18	127	96	45.4% (103/227)	36.3% (87/240)	0.28 (0.23–0.35)	0.24 (0.20–0.30)
Pakistan	8.49	22.37	163	264	63.9% (124/194)	74.6% (206/276)	0.32 (0.27–0.38)	0.89 (0.77–1.02)
Peru	7.59	21.61	95	118	33.7% (83/246)	34.3% (104/303)	0.20 (0.16–0.25)	0.28 (0.23–0.34)
Tanzania	0.45	21.36	263	173	73.0% (173/237)	58.7% (152/259)	0.68 (0.58–0.79)	0.54 (0.46–0.63)
South Africa	0.94	20.71	28	57	12.9% (27/209)	19.0% (56/295)	0.06 (0.04–0.08)	0.13 (0.10–0.16)

a Across the 8 MAL-ED sites, 36.9% (781/2116) of children experienced at least one persistent *Campylobacter* episode, with Tanzania and Bangladesh experiencing the highest incidence rates.

Across the eight sites, 45.5% (781/1715) of children experienced at least one persistent *Campylobacter* episode, with Tanzania (73.3% (173/237)) having the highest proportion, followed by Bangladesh (69.0% (145/210)) and Pakistan (63.9% (124/194). This ranking shifted based on ELISA results, but the same sites were identified (Table 1) as the top three with the highest burden. Most children (32.6% (560/1715)) experienced only a single persistent episode, with the rest (12.9% (221/1715)) experiencing two or more persistent episodes. Bangladesh, Tanzania, and Pakistan had the highest incidence rates of persistent episodes, with Pakistan leading with 0.89 (95% CI: 0.77–1.02) persistent episodes per child year of follow-up.

All *Campylobacter* positive episodes per qPCR and ELISA separately, for the eight study sites, are visualized as sequence plots in Supplementary Figure S1. The distribution of the total duration of *Campylobacter* positivity is presented in Supplementary Figure S2.

### Risk factors associated with persistent *Campylobacter* episodes

The odds of experiencing a persistent *Campylobacter* episode throughout 24 months of follow-up increased by 22% (OR = 1.18, 95% Confidence Internal (CI): 1.16; 1.29) and 17% (OR = 1.17; 95% CI: 1.09; 1.25) with each additional prior bacterial and protozoal infection, respectively (Table 2). However, the number of prior diarrhea episodes displayed a protective effect (OR: 0.84; 95% CI: 0.79; 0.90), with decreased odds of having a persistent *Campylobacter* infection with every additional diarrheal episode. No other covariate was associated with persistent *Campylobacter* infections. Unadjusted and adjusted logistic regressions are presented in Table 2.

**Table 2:** Unadjusted and Adjusted associations between the occurrence of persistence episodes and sociodemographic and household characteristics, across the eight MAL-ED sites.

	Unadjusted			Adjusted^a^		
	Odds ratio	95% CI	p-value	Odds ratio	95% CI	p-value
**Socio demographic characteristics**						
Sex						
Male	[Ref]	[Ref]	[Ref]	[Ref]	[Ref]	[Ref]
Female	1.08	0.90–1.28	0.397	1.25	0.98–1.59	0.069
Birth Weight (Kg)	0.86	0.78–0.95	**0.002**	1.37	0.94–1.99	0.103
Baseline LAZ	0.95	0.87–1.03	0.255	1.16	0.97–1.38	0.095
LAZ at 24 months of age	0.64	0.59–0.70	**<0.001**	0.76	0.62–0.92	**0.006**
Baseline WAZ	0.88	0.81–0.94	**<0.001**	0.98	0.82–1.17	0.828
WAZ at 24 months of age	0.67	0.61–0.73	**<0.001**	0.92	0.76–1.11	0.379
Number of diarrhea episodes by 2nd year of life	1.09	1.06–1.14	**<0.001**	0.84	0.79–0.90	**<0.001**
Number of bacterial infections by 2nd year of life^b^	1.25	1.22–1.28	**<0.001**	1.22	1.16–1.29	**<0.001**
Number of protozoal infections by 2nd year of life	1.35	1.30–1.40	**<0.001**	1.17	1.09–1.25	**<0.001**
Number of viral infections by 2nd year of life	1.21	1.18–1.25	**<0.001**	1.05	0.98–1.12	0.134
Maternal education						
None	[Ref]	[Ref]	[Ref]	[Ref]	[Ref]	[Ref]
Primary complete	0.73	0.54–0.97	0.031	0.93	0.58–1.50	0.675
Secondary complete	0.46	0.32–0.59	**<0.001**	0.98	0.60–1.60	0.828
Higher education complete	0.24	0.17–0.35	**<0.001**	1.12	0.58–2.18	0.744
**WAMI score** (Mean (SD))	0.06	0.04–0.09	**<0.001**	0.35	0.10–1.26	0.108
**Household characteristics**						
Household occupancy						
0–2 people	[Ref]	[Ref]	[Ref]	[Ref]	[Ref]	[Ref]
2–3 people	1.72	1.17–2.55	**0.006**	1.22	0.73–2.06	0.437
3–4 people	2.34	1.60–3.43	**<0.001**	1.33	0.79–2.25	0.279
>4 people	2.54	1.75–3.68	**<0.001**	1.14	0.67–1.94	0.623
Water source						
Unimproved	[Ref]	[Ref]	[Ref]	[Ref]	[Ref]	[Ref]
Improved	0.45	0.35–0.60	**<0.001**	1.26	0.73–2.20	0.409
Sanitation						
Open defecation/Unimproved	[Ref]	[Ref]	[Ref]	[Ref]	[Ref]	[Ref]
Improved	0.87	0.72–1.05	0.134	0.84	0.55–1.29	0.427
Floor material						
Natural/Rudimentary	[Ref]	[Ref]	[Ref]	[Ref]	[Ref]	[Ref]
Finished	0.47	0.40–0.57	**<0.001**	0.75	0.48–1.17	0.199
Walls						
Natural/Rudimentary	[Ref]	[Ref]	[Ref]	[Ref]	[Ref]	[Ref]
Finished	0.50	0.41–0.59	**<0.001**	1.15	0.74–1.80	0.534
Roof						
Natural/Rudimentary	[Ref]	[Ref]	[Ref]	[Ref]	[Ref]	[Ref]
Finished	0.79	0.64–0.97	**0.024**	1.16	0.81–1.65	0.534
Poultry ownership						
No	[Ref]	[Ref]	[Ref]	[Ref]	[Ref]	[Ref]
Yes	1.69	1.40–2.03	**<0.001**	1.36	0.99–1.87	**0.051**
Cattle ownership						
No	[Ref]	[Ref]	[Ref]	[Ref]	[Ref]	[Ref]
Yes	2.01	1.62–2.50	**<0.001**	1.07	0.65–1.76	0.801

Adjusted associations between the occurrence of a persistent *Campylobacter* episode and sociodemographic, anthropometric, diagnostic, and household characteristics, modelled by a multivariate logistic regression model.

The odds of having a persistent *Campylobacter* episode throughout 24 months of follow-up increased by 22%, and 17% with every additional bacterial and protozoal infection, respectively, by the end of follow-up. There is a decreased odds of having a persistent *Campylobacter* infection with every additional diarrheal episode. Bold: p < 0.05.

a Adjusted for all variables plus Site with fixed effects.

b Excluding *Campylobacter* spp.

### Anthropometric consequences of *Campylobacter* persistent episodes

**Fig. 1:**
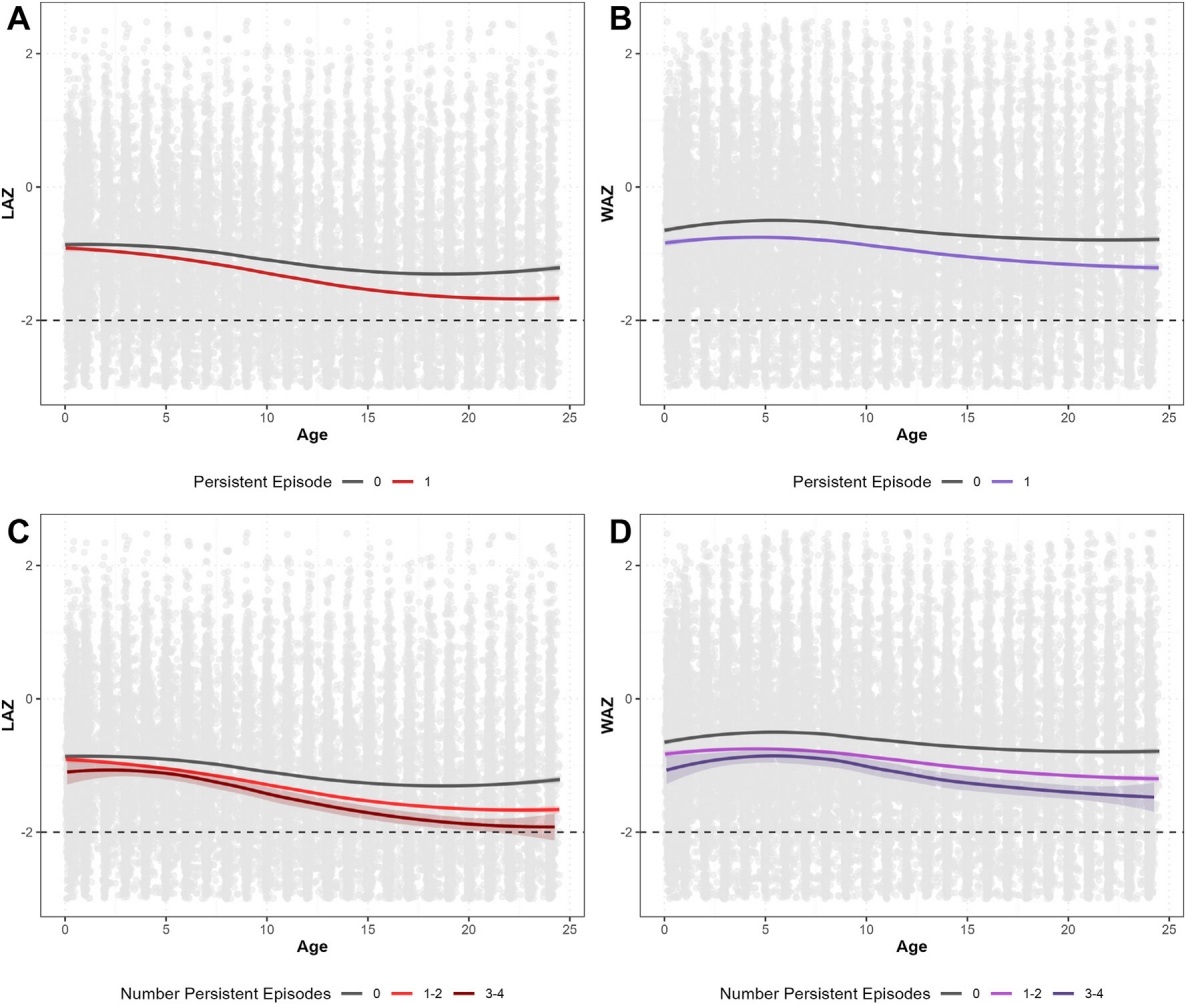
**Growth Patterns in Children with and without Persistent *Campylobacter* Episodes. [A] Length for Age Z-score (LAZ) and [B] Weight for Age Z-score comparing children who had at least one persistent *Campylobacter* episode (1) to children who did not (0). [C] Length for Age Z-score (LAZ) and [D] Weight for Age Z-score comparing children who had multiple persistent *Campylobacter* episodes (1**–**2; 3**–**4) to children who did not (0), across all eight MAL-ED study sites**. At birth children with and without persistent infection appear nearly identical, but differences in linear and ponderal growth led to progressive differences in age adjusted length and weight over the period of study. These differences are greater among children who experienced more than one persistent *Campylobacter* episode.

Growth patterns in children with and without persistent *Campylobacter* episodes as defined by qPCR diagnostics are portrayed in Fig. 1. Children who experienced a persistent *Campylobacter* episode had an attained 24-month LAZ score that was 0.23 (95% CI: −0.31, −0.15) less than children who did not experience a *Campylobacter* persistent episode (Table 3). The same model estimated that the number of bacterial and protozoal infections was statistically significantly associated with decreased LAZ scores at 24 months of age, although effects were marginal and an order of magnitude less. Unadjusted and adjusted linear regressions are presented in Table 3.

**Table 3:** Unadjusted and adjusted associations between the LAZ scores at 24 months of age and the occurrence of persistent *Campylobacter* episodes, across the eight MAL-ED sites.

	Unadjusted			Adjusted^a^		
	LAZ score at 24 months	95% CI	p-value	LAZ score at 24 months	95% CI	p-value
Persistent *Campylobacter* episodes	−0.58	−0.69; −0.48	**<0.001**	−0.23	−0.31; −0.15	**<0.001**
**Socio demographic characteristics**						
Sex						
Male	[REF]	[REF]	[REF]	[REF]	[REF]	[REF]
Female	0.28	0.07; 0.28	**0.001**	0.12	0.05; 0.19	**0.001**
Birth Weight (Kg)	0.20	0.14; 0.26	**<0.001**	−0.14	−0.21; −0.07	**<0.001**
Baseline LAZ	0.64	0.61; 0.68	**<0.001**	0.50	0.46; 0.54	**<0.001**
Baseline WAZ	0.45	0.42; 0.49	**<0.001**	0.12	0.07; 0.16	**<0.001**
Number of diarrhea Episodes by 2nd year of life^b^	−0.03	−0.05; −0.01	**0.002**	0.00	−0.01; 0.02	0.672
Number of bacterial infections by 2nd year of life	−0.07	−0.08; −0.06	**<0.001**	−0.03	−0.04; −0.01	**<0.001**
Number of protozoal infections by 2nd year of life	−0.12	−0.14; −0.10	**<0.001**	−0.03	−0.05; −0.01	**0.002**
Number of viral infections by 2nd year of life	−0.06	−0.08; −0.05	**<0.001**	**0.02**	**0.00; 0.04**	**0.030**
Maternal education						
None	[REF]	[REF]	[REF]	[REF]	[REF]	[REF]
Primary complete	0.33	0.15; 0.50	**<0.001**	0.07	−0.06; 0.19	0.304
Secondary complete	0.52	0.37; 0.67	**<0.001**	0.03	−0.11; 0.17	0.674
Higher education complete	1.14	0.94; 1.34	**<0.001**	0.12	−0.06; 0.30	0.198
**WAMI Score** (Mean (SD))	2.32	2.11; 2.53	**<0.001**	0.85	0.51; 1.20	**<0.001**
**Household Characteristics**						
Household occupancy						
0–2 people	[REF]	[REF]	[REF]	[REF]	[REF]	[REF]
2–3 people	1.18	−0.03; 0.38	0.089	0.15	0.11; 0.29	**0.035**
3–4 people	−0.1	−0.30; 0.10	0.341	0.12	−0.02; 0.26	0.103
>4 people	−0.21	−0.40; −0.01	**0.038**	0.06	−0.08; 0.21	0.377
Water source						
Unimproved	[REF]	[REF]	[REF]	[REF]	[REF]	[REF]
Improved	0.81	0.64; 0.97	**<0.001**	−0.12	−0.28; −0.04	0.129
Sanitation						
Open defecation/Unimproved	[REF]	[REF]	[REF]	[REF]	[REF]	[REF]
Improved	0.79	0.69; 0.90	**<0.001**	−0.02	−0.14; 0.11	0.776
Floor material						
Natural/Rudimentary	[REF]	[REF]	[REF]	[REF]	[REF]	[REF]
Finished	0.51	0.41; 0.62	**<0.001**	0.09	−0.03; 0.22	0.153
Walls						
Natural/Rudimentary	[REF]	[REF]	[REF]	[REF]	[REF]	[REF]
Finished	0.54	0.44; 0.65	**<0.001**	−0.03	−0.15; 0.10	0.683
Roof						
Natural/Rudimentary	[REF]	[REF]	[REF]	[REF]	[REF]	[REF]
Finished	0.47	0.35; 0.59	**<0.001**	−0.09	−0.19; 0.01	0.093
Poultry ownership						
No	[REF]	[REF]	[REF]	[REF]	[REF]	[REF]
Yes	−0.43	−0.54; −0.32	**<0.001**	0.00	−0.09; 0.09	0.971
Cattle ownership						
No	[REF]	[REF]	[REF]	[REF]	[REF]	[REF]
Yes	−0.42	−0.65; −0.39	**<0.001**	−1.01	−0.18; 0.06	0.314

Adjusted association between LAZ score attained at 24 months of age and the occurrence of a persistent *Campylobacter*, modelled by a multivariable linear regression model.

Children who experienced a persistent *Campylobacter* episode had an overall LAZ score by 24 months of age of 0.23 (95% CI: −0.31, −0.15) less than children who did not experience a *Campylobacter* persistent episode. Bold: p < 0.05.

a Adjusted for all variables plus Site with fixed effects.

b Excluding *Campylobacter* spp.

Associations between symptomatic and asymptomatic *Campylobacter*, weight gain over 3-months, and change in length over 9-months, are shown in Table 4. Table 4 additionally shows results from a prior published analysis of acute *Campylobacter* infections and short-term growth among 0–6-year-old Peruvian children for comparison.^23^ Children who experienced an asymptomatic *Campylobacter* episode put on 18.6 g (95% CI: −37.3, 0.0) less weight over the 3-month intervals compared to children who did not have an asymptomatic episode. Children who experienced a symptomatic *Campylobacter* episode put on 59.2 g (95% CI: −42.2, −6.0) less weight over the intervals compared to children who did not. Each symptomatic *Campylobacter* episode was also associated with a non-significant 0.10 cm (95% CI: −0.209, 0.008) less gain in length over a 9-month period. Asymptomatic *Campylobacter* detections were not statistically significantly associated with poorer growth.

**Table 4:** Adjusted associations between symptomatic and asymptomatic *Campylobacter* infection of any duration and change in weight and length gain over 3-month and 9-months period, respectively.

	Current analysis^a^	MAL-ED Peru only	Lee et al. 2013
9-month change in length (cm) models			
Asymptomatic *Campylobacter*	0.219 (−0.140, 0.577)	0.957 (−0.295, 2.209)	−0.01 (−0.09, 0.07)
Symptomatic *Campylobacter*	−0.100 (−0.209, 0.008)	−0.024 (−0.229, 0.182)	−0.06 (−0.12, 0.01)
Non-*Campylobacter* diarrhea	−0.025 (−0.101, 0.051)	−0.017 (−0.146, 0.111)	−0.04 (−0.06, −0.02)
3-month change in weight (grams) models			
Asymptomatic *Campylobacter*	−18.9 (−37.6, −0.2)	−19.8 (−6.7, 2.7)	−65.5 (−128.0, −3.0)
Symptomatic *Campylobacter*	−59.2 (−42.2, −6.0)	−50.6 (−10.7, 5.6)	−43.9 (−87.6, −0.1)
Non-*Campylobacter* diarrhea	−24.1 (−42.2, −6.0)	−10.9 (−38.7, 17.0)	−22.9 (−35.3, −10.1)

a Adjusted for site with fixed effects and random intercept, socio-economic status (WAMI score), prior nutritional status, sex, birthweight, episodes of diarrheal disease that were not associated *Campylobacter* in the same period, presence of non-*Campylobacter* pathogens detected over the same period.

Among children who had at least one episode of *Campylobacter*, the association between persistent episodes and poorer weight gain (−28.7, 95% CI: −63.4 g, 6.0 g) over a 3-month window compared to children with episodes that resolved within 31 days was not statistically significant (Table 5). Persistent *Campylobacter* carriage was however associated with −0.13 cm poorer linear growth (95% CI: −0.25, −0.02) over a 9-month period compared to children with an episode that resolved within 31 days.

**Table 5:** Adjusted association between persistent *Campylobacter* episodes and change in weight and length gain over 3-month and 9-months period, respectively.

Length of *Campylobacter* episode^a^	Weight gain (grams/3 months)	Length gain (cm/9 mo)
1–31-day episode	*Ref*	*Ref*
32–89-day episode	−20.3 (−49.4, 8.8)	−0.123 (−0.222, −0.023)
90+ day episode (persistent)	−28.7 (−63.4, 6.0)	−0.134 (−0.246, −0.022)

Persistent episodes of increased duration are associated with greater magnitudes of linear and ponderal adverse effects on growth.

a Adjusted for site with fixed effects and random intercept, socio-economic status (WAMI score), prior nutritional status, sex, birthweight, episodes of diarrheal disease that were not associated *Campylobacter* in the same period, presence of non-*Campylobacter* pathogens detected over the same period.

## Discussion

Persistent and/or recurrent *Campylobacter* infections are common among children living in diverse, resource constrained settings, and these persistent infections have a stronger association with reduced linear growth and weight gain than shorter episodes, even in the absence of diarrhea. Given that relapse and/or chronic carriage is a common phenomenon among children infected with *Campylobacter*, with or without diarrhea, current clinical guidelines, which do not recommend the treatment of asymptomatic *Campylobacter*, or any retesting after treatment, may need to be reconsidered. Certainly, those with *Campylobacter* infections should be advised of a risk or prolonged or relapsing disease given our findings, combined with that of others.^17^, ^18^, ^19^, ^20^^,^^29^^,^^30^

Associations between symptomatic *Campylobacter* and short-term changes in growth were similar to our prior report of an older, Peruvian cohort: −0.10 cm less growth over 9-months per symptomatic *Campylobacter* episode, vs −0.06 cm less growth over the same time period in the prior results.^23^ This estimate is similar to culture positive shigellosis in a prior study in Peru, which estimated 0.081 cm less linear growth over the subsequent 9-month period.^60^ Estimates of the association between asymptomatic and symptomatic *Campylobacter* detections and weight were also similar or somewhat greater than our previous analysis. For example, a symptomatic episode was associated with 60.8 g less weight gain in this study, compared to 43.9 g less weight gain in a prior Peruvian pediatric cohort, while an asymptomatic episode was associated with 18.6 g less weight gain compared to 65.5 g in the prior cohort. These differences may result from differences in the method of *Campylobacter* detection. In the prior study,^23^ culture-based identification of *Campylobacter* was employed and here, molecular identification was employed. Thus, the prevalence of *Campylobacter* was not directly comparable between the two studies with more sensitive molecular diagnostics likely detecting more mild or light infections in this study that have more attenuated effects on growth.^61^

When comparing children with persistent vs short episodes of *Campylobacter*, an episode of 90 or more days in duration was associated with less weight gain and cm less linear growth compared to children with shorter episodes. The finding that children with persistent *Campylobacter* episodes gained less weight, and experienced less linear growth, during the periods when they were infected, complements analysis that by 24 months of age, children who experienced persistent *Campylobacter* on average remained smaller. In combination with the analysis of risk factors for *Campylobacter*, our results suggest that associations between attained length and prior persistent *Campylobacter* may have been driven both by greater risk for *Campylobacter* among children with smaller length-for-age and by poorer growth during persistent episodes. Animal studies have also demonstrated that persistent *Campylobacter* is a cause of growth faltering and smaller attained size.^62^, ^63^, ^64^ However, it is also possible that persistent *Campylobacter* carriage in children may be a marker of enteropathy rather than the direct cause of growth failure.

The observed persistence of *Campylobacter* could be due to the result of at least four underlying determinants, or a combination of these factors. The first is that the existence of microbial features that enable host adaptation and prolonged carriage may be present in some of these isolates. Molecular mechanisms of *Campylobacter* persistence in the human gut have been studied and hypothesized to be associated with molecular mimicry of gangliosides by sialylated *C. jejuni* lipo-oligosaccharides,^65^ lysosome evasion within epithelial cells,^66^ its capacity to enter a viable but non culturable state,^67^ and its biofilm production capacity.^68^^,^^69^ A recent human challenge study designed to evaluate the prophylactic efficacy of rifaximin identified recrudescent *Campylobacter* infections in five participants,^29^^,^^70^ all of which were screened for immunosuppression prior to enrollment. Comparison of gene variants between acute and recrudescent infections showed that the latter had an increased number of genomic variants, that were hypothesized to be a result of either secondary infections or within host evolution within the gut. Genes associated with recurrent infections included the cell invasion protein A (*cipA*) which presumably was associated with cellular invasion and persistence through flagellar modification. CmeR, the transcriptional repressor of the *cmeABC* operon, was also associated with relapsed infections. The CmeABC is a chromosomal drug efflux pump composed of a periplasmic fusion protein (CmeA), an inner membrane transporter (CmeB), and an outer membrane protein (CmeC), while CmeR binds to the inverted repeats of the promoter region of the operon.^71^, ^72^, ^73^ Single base changes in the *cmeR* gene or the inverted repeat region results in an altered binding site and thus overexpression of the CmeABC efflux pump, and thus enhanced resistance to bile salts and a variety of antimicrobials.^72^^,^^74^, ^75^, ^76^ Future work should aim to better understand how these factors may relate to the risk of persistent infection.

Alternatively, host immunosuppression as the result of undernutrition and environmental enteropathy may induce an immunotolerant state resulting in decrease immune responsiveness to enteropathogens akin to the hyporesponsiveness to oral vaccines.^77^^,^^78^ In murine models zinc deficiency and the use of antibiotics both increase the amount of inflammation associated with *Campylobacter* and increase the time to pathogen clearance.^79^ Human GWAS studies have revealed two features of innate resistance that are protective against *Campylobacter* diarrhea, Rho Guanine Nucleotide Exchange Factor 10 gene (ARHGEF10) and CLN8 a transmembrane protein that localizes to the endoplasmic reticulum which appears to be involved with the trafficking of the cytolethal -distending toxin secreted by *Campylobacter* species and innate differences in vulnerability may be involved in determining the duration of infection. Lastly, in this environment of heavy fecal contamination multiple detections, even a pattern of continuous infection may be mimicked by multiple recurrent infections. This has been explored to a limited extent in the MAL-ED study and others.^80^^,^^81^ Multiple discreet *Campylobacter* infections may be associated may be associated with growth faltering, limiting the ability of children to catch up in comparison to children who only experience a single infection.^57^ Clarity on the relative importance of persistent infection relative to frequent reinfection will require longitudinal samples that allow for highly discriminant genomic typing, which have not yet been reported in the literature but are of clear importance to understand how to intervene to diminish the impact of persistently detected *Campylobacter* in young children.

It should be noted that a prior study of this cohort^8^ found detrimental effects of *Campylobacter* infection episodes of all durations on growth but failed to find significant effects of persistent *Campylobacter* infections specifically. The inconsistency with results presented here is due to the infrequent sample testing by ELISA (limited to quarterly in the second year of life compared to monthly in the current analysis of qPCR results). This resulted in an analysis of insufficient temporal resolution to fully capture persistent episodes with 28.9% of participants identified as having persistent infection in the prior analysis compared with 36.9% of subjects in the current analysis.

Persistent symptomatic diarrhea in children living in resource constrained settings has been described,^82^^,^^83^ but should be recognized as distinct from the phenomenon of persistent infections, frequently asymptomatic, that is the topic of this work. Persistent diarrhea is a clinical syndrome of diarrhea lasting greater than 14 days.^82^ Serial cultures and diagnostics reveal that the episode is not caused by persistent infections but rather multiple serial infections in undernourished children and the incidence of persistent diarrhea has been in decline over the past 30 years.^84^ Even when *Campylobacter* infections persist, they rarely cause symptomatic illness of 14 days duration, and they are no more likely to occur in persistent than in acute diarrhea.^85^

This study does have several limitations as well as strengths. This study was not designed to study persistent episodes of *Campylobacter* and the study's sampling strategy could have been more informative if it was more frequent. Consequentially, the results should be interpreted with caution as the study was not powered to detect the reported growth outcomes. Strengths of the study are the diverse study sites that provide a practical balance between internal validity and generalizability of findings. Furthermore, the study tested a very large number of stool samples with state-of-the-art diagnostic methodologies under a common protocol in highly diverse epidemiologic settings and human populations. Finally, for the size of the study and intensity of sampling events, the proportion of missing data is considered low and such a strength of the current analysis.

Our study supports the observation of the human challenge study demonstrating that persistence of *Campylobacter* infection is both common and carries demonstrable unfavorable health consequences. Clinicians and patients should be aware of the possibility of disease recrudescence following adequate therapy to minimize delayed health care seeking.

## Contributors

Study design: AAML, CJM, TA, GK, EM, AS, AZ, JL, MPO, PPY, ERH, MNK; Funding acquisition: AAML, CJM, TA, GK, EM, AS, AZ, JL, ERH, MNK; Conceptualization: FS, JMC, MPO, PPY, EM, BP, KKC, CTP, GOL, MNK; Investigation: FS, JMC, MPO, PPY, EM, BP, KKC, CTP, GOL, MNK; Writing original draft: FSS, JMC, GOL, MNK; Writing review & editing: FSS, JMC, GOL, AAML, CJM, TA, GK, EM, AS, AZ, JL, ERH, EM, BP, CTP, KKC, PPY, MPO, MNK; Methodology: FSS, JMC, GOL, MNK; Formal Analysis: FSS, JMC, GOL, MNK. All authors had access to the data and accept responsibility for submitting the article for publication.

## Data sharing statement

De-identified data from the MAL-ED cohort study is available at ClinEpi DB (https://clinepidb.org/ce/app).

## Declaration of interests

AKZ is currently employed at the Bill and Melinda Gates Foundation as the President of Gender Equality. The Gates Foundation was the original funder of this project. They have not had a role in this analysis.
