# Supplementary material for: The epidemiology and impact of persistent Campylobacter infections on childhood growth among children 0–24 months of age in resource-limited settings
Source: eClinicalMedicine. 2024 Sep 28;76:102841. doi: 10.1016/j.eclinm.2024.102841 (PMC11460251; doi:10.1016/j.eclinm.2024.102841)
Supplement: Supplementary File [file mmc1.pdf]

Supplementary File 1 - Supplementary Methods

Sample collection and storage. Stool samples were collected and transported on cold chain before being aliquoted and stored at -80°C. The duration of storage ranged from two to four years before testing with qPCR at each of the local labs in the participant countries.

Nucleic acid extraction. Nucleic acid was extracted with the manual QIAamp Fast DNA Stool mini kit (Qiagen, Hilden, Germany) with pretreatment steps that included bead beating (1). Briefly, 200 mg of raw stool was first lysed with QIAamp Inhibitex buffer, beaten for 2 min with 212 to 300-μm glass beads (Sigma, St. Louis, MO), and incubated at 95°C for 5 min. The samples were centrifuged at full speed for 1 min to pellet stool particles, then 600 μl of lysate were extracted and eluted in 200 μl of elution buffer following the manufacturer's instructions. Two extrinsic controls, Phocine Herpesvirus (PhHV) and bacteriophage MS2, were spiked into lysis buffer to monitor extraction and amplification efficiency. One extraction blank was included per batch of extraction to rule out lab contamination.

qPCR testing. TaqMan Array Card (TAC, Thermo Fisher) is a microfluidic card compartmentalizing 384 individual TaqMan probe based real time PCR reactions. Gene targets and PCR assays were adapted from publications whenever possible, with modifications if needed to the primers and probes to optimize their performance under the universal TAC cycling condition. The standard primer and probe concentrations were 900 nM and 250 nM, respectively. The performance of the assays was evaluated for linearity, intra-assay precision, inter-assay precision, limit of detection, and specificity as outlined previously (1-3). *Campylobacter* spp. was detected based on a 60kDa chaperonin, *Cpn60* targeting an 86 bp region (1), with the primers and probes including Cpn60_Fw1 (AF461064:499-521): AAAGTIGGMAAAGATGGTGTTAT, Cpn60_Fw2: AAAGTIGGWAAAGACGGYGTTAT, Cpn60_Rv1 (AF461064:565-584): TCAAATTGCATACCYTCAAC, Cpn60_Probe1 (AF461064 523-539): TTTGCCTCTTCMACAGT, Cpn60_Probe2: TTTGCTTCTTCWACAGT. 20 μl of nucleic acid extract was mixed with 50 μl of AgPath-ID One-Step RT-PCR buffer (Thermo Fisher), 4 μl of enzyme mix, and nuclease free water to a 100-μl final volume. The cycling conditions consisted reverse transcription step at 45°C for 20 minutes, the initial denaturation at 95°C for 10 minutes, followed by 40 cycles of 95°C for 15 seconds and 60°C for 1 minute. The reaction mixture underwent quantitative PCR on ViiA 7, or QuantStudio 7, or QuantStudio 12K Flex systems (Thermo Fisher). The results were processed and exported from QuantStudio Real Time PCR Software (Thermo Fisher).

qPCR result interpretation. Quantification cycle (Cq) was determined with a baseline threshold algorithm, where fluorescence from amplification exceeded the background. A cutoff of 35 was applied for positivity as the averaged Cq values of the targets at lower limit of detection. Positive results were considered valid only when the corresponding extraction blank was negative for the relevant target; negative results were considered valid only when the extrinsic controls were positive for the given samples. Data flagged by the real time PCR software, i.e. BADROX in combination with NOISE or SPIKE, were also excluded for data quality. The invalidated results were excluded from analysis. Combining the above quality controls, the validity rate of the entire testing was 94.8%.

1. Liu J, Gratz J, Amour C, et al. Optimization of Quantitative PCR Methods for Enteropathogen Detection. PLoS One 2016; 11(6): e0158199.
2. Liu J, Gratz J, Amour C, et al. A laboratory-developed TaqMan Array Card for simultaneous detection of

19 enteropathogens. J Clin Microbiol 2013; 51(2): 472-80.
3. Liu J, Kabir F, Manneh J, et al. Development and assessment of molecular diagnostic tests for 15 enteropathogens causing childhood diarrhoea: a multicenter study. Lancet Infect. Dis. 2014; 14(8): 716-24.

ELISA testing. ELISA was done on stools that were frozen at -70°C without preservatives or transport media. 0.3 gm of stool was added to 0.6 ml bacterial specimen diluent and mixed and added to the plate with positive and negative controls. The microplate was then covered and incubated at 20-25°C for 1 hour at which point they were emptied and washed 3 times with diluted wash buffer. Enzyme conjugate (200 µl) was then added to each well and the plate was covered and incubated at 20-25° C for 30 minutes. The plate was then emptied, washed 5 times with wash buffer and 200 µl of color substrate was added and the plate was covered and incubated at 20-25°C for 10 minutes. 50 ul of stop solution was added to each well and the plate was read within 10 minutes and read at 450 nm. Samples from plates yielding optical densities of > 0.170 with in range positive and negative controls were interpreted as positive.

Table 1.
MIQE checklist for authors, reviewers, and editors.[1]

Item to check	Importance	Item to check	Importance
Experimental design		qPCR oligonucleotides	
Definition of experimental and control groups	E P5, Ref.1-2	Primer sequences	E P6
Number within each group	E P10	RTPrimerDB identification number	D
Assay carried out by the core or investigator's laboratory?	D	Probe sequences	D[4] P6
Acknowledgment of authors' contributions	D	Location and identity of any modifications	E Supplemental
Sample		Manufacturer of oligonucleotides	D Supplemental
Description	E P5, Ref.2	Purification method	D
Volume/mass of sample processed	D P5, Ref.2	qPCR protocol	
Microdissection or macrodissection	E NA	Complete reaction conditions	E Supplemental
Processing procedure	E Supplemental	Reaction volume and amount of cDNA/DNA	E Supplemental
If frozen, how and how quickly?	E Supplemental	Primer, (probe), Mg^{2+}, and dNTP concentrations	E Supplemental
If fixed, with what and how quickly?	E NA	Polymerase identity and concentration	E P6, supplemental
Sample storage conditions and duration (especially for FFPE[2] samples)	E Supplemental	Buffer/kit identity and manufacturer	E P6, supplemental
Nucleic acid extraction		Exact chemical composition of the buffer	D
Procedure and/or instrumentation	E P5,supplemental	Additives (SYBR Green I, DMSO, and so forth)	E NA
Name of kit and details of any modifications	E Supplemental	Manufacturer of plates/tubes and catalog number	D
Source of additional reagents used	D	Complete thermocycling parameters	E Supplemental
Details of DNase or RNase treatment	E NA	Reaction setup (manual/robotic)	D Supplemental
Contamination assessment (DNA or RNA)	E Supplemental	Manufacturer of qPCR instrument	E Supplemental
Nucleic acid quantification	E NA	qPCR validation	
Instrument and method	E Supplemental	Evidence of optimization (from gradients)	D
Purity (A_{260}/A_{280})	D	Specificity (gel, sequence, melt, or digest)	E Ref.3-5
Yield	D	For SYBR Green I, C_q of the NTC	E NA
RNA integrity: method/instrument	E NA	Calibration curves with slope and *y* intercept	E Ref.3-5
RIN/RQI or C_q of 3′ and 5′ transcripts	E NA	PCR efficiency calculated from slope	E Ref.3-5
Electrophoresis traces	D	CIs for PCR efficiency or SE	D
Inhibition testing (C_q dilutions, spike, or other)	E P5, supple-mental	r^2 of calibration curve	E Ref.3-5
Reverse transcription		Linear dynamic range	E Ref.3-5
Complete reaction conditions	E P6,supple-mental	C_q variation at LOD	E Ref.3-5
Amount of RNA and reaction volume	E NA	CIs throughout range	D
Priming oligonucleotide (if using GSP) and concentration	E Supplemental	Evidence for LOD	E Ref.3-5
Reverse transcriptase and concentration	E NA	If multiplex, efficiency and LOD of each assay	E Ref.3-5
Temperature and time	E Supplemental	Data analysis	
Manufacturer of reagents and catalogue numbers	D Supplemental	qPCR analysis program (source, version)	E Supplemental
C_qs with and without reverse transcription	D[3]	Method of C_q determination	E Supplemental
Storage conditions of cDNA	D	Outlier identification and disposition	E NA
qPCR target information		Results for NTCs	E Supplemental
Gene symbol	E P5	Justification of number and choice of reference genes	E Ref.3-5
Sequence accession number	E NA	Description of normalization method	E NA
Location of amplicon	D	Number and concordance of biological replicates	D
Amplicon length	E NA	Number and stage (reverse transcription or qPCR) of technical replicates	E NA
In silico specificity screen (BLAST, and so on)	E Ref.3-5	Repeatability (intraassay variation)	E Ref.3-5
Pseudogenes, retropseudogenes, or other homologs?	D	Reproducibility (interassay variation, CV)	D Ref.3-5
Sequence alignment	D	Power analysis	D
Secondary structure analysis of amplicon	D	Statistical methods for results significance	E P6-7
Location of each primer by exon or intron (if applicable)	E NA	Software (source, version)	E Ref.3-5
What splice variants are targeted?	E NA	C_q or raw data submission with RDML	D

REFERENCES

1. The MAL-ED study: a multinational and multidisciplinary approach to understand the relationship between enteric pathogens, malnutrition, gut physiology, physical growth, cognitive development, and immune responses in infants and children up to 2 years of age in resource poor environments. Clin Infect Dis 2014; 59 Suppl 4: S193-206.

2. Rogawski ET, Liu J, Platts-Mills JA, et al. Use of quantitative molecular diagnostic methods to investigate the effect of enteropathogen infections on linear growth in children in low-resource settings: longitudinal analysis of results from the MAL-ED cohort study. Lancet Glob Health 2018; 6(12): e1319-e28.

3. Liu J, Gratz J, Amour C, et al. Optimization of Quantitative PCR Methods for Enteropathogen Detection. PLoS One 2016; 11(6): e0158199.

4. Liu J, Gratz J, Amour C, et al. A laboratory-developed TaqMan Array Card for simultaneous detection of 19 enteropathogens. J Clin Microbiol 2013; 51(2): 472-80.

5. Liu J, Kabir F, Manneh J, et al. Development and assessment of molecular diagnostic tests for 15 enteropathogens causing childhood diarrhoea: a multicenter study. Lancet Infect. Dis. 2014; 14(8): 716-24.
