# Supplementary material for: The epidemiology and impact of persistent Campylobacter infections on childhood growth among children 0–24 months of age in resource-limited settings
Source: eClinicalMedicine. 2024 Sep 28;76:102841. doi: 10.1016/j.eclinm.2024.102841 (PMC11460251; doi:10.1016/j.eclinm.2024.102841)
Supplement: Supplementary Figures and Tables [file mmc2.docx]

**Supplementary Figure 1 and Supplementary Figure 2**

**
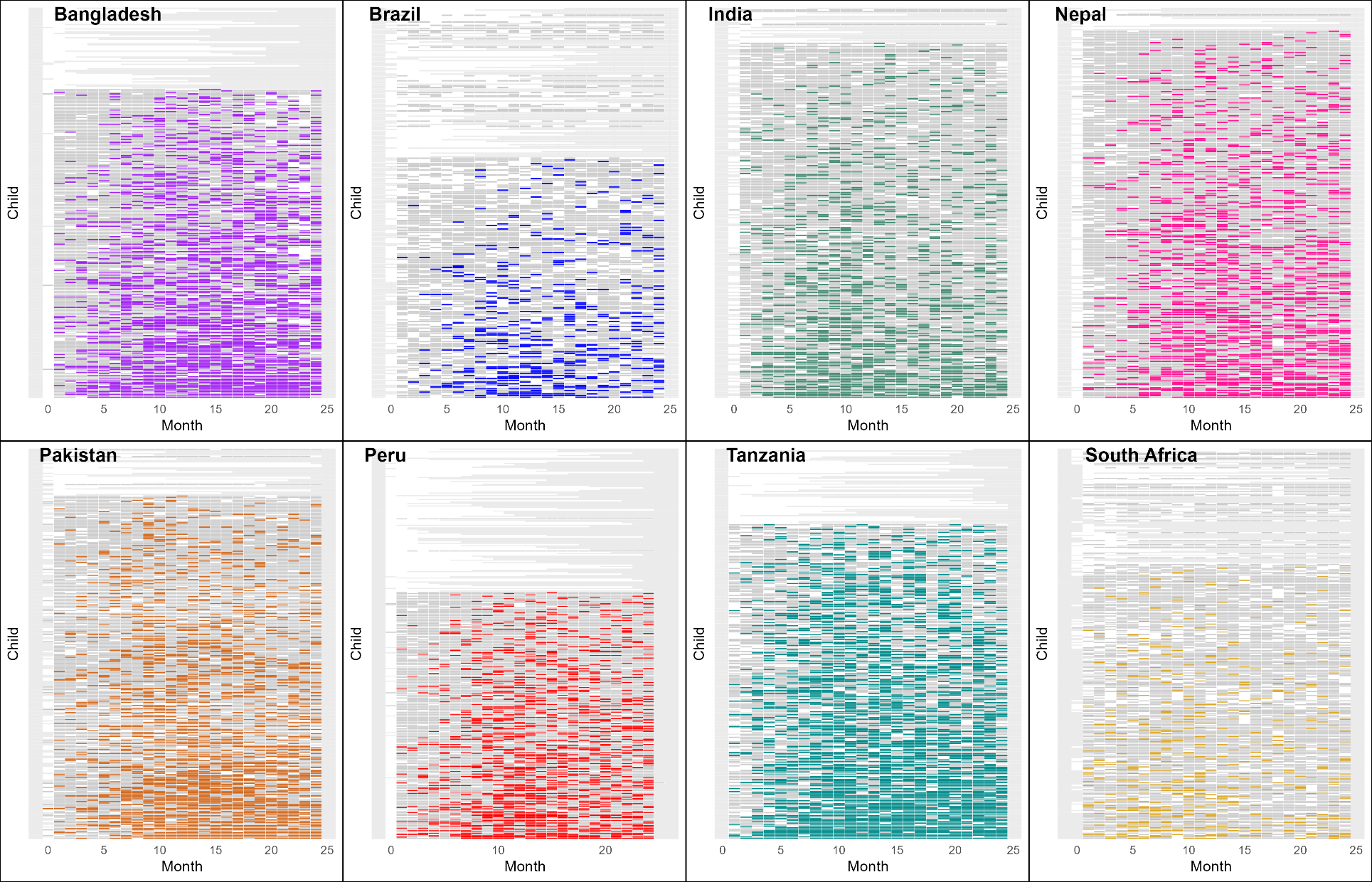
Supplementary Figure 1.** **Sequence plot showing *Campylobacter* positive samples by TAC across follow-up.** Each child is represented on the vertical axis. The ***Campylobacter*** status is positive if the corresponding child month is positive, gray if negative, and white if no sample was obtained that month. Plots are organized by density of child-infection months, and towards the bottom of each counties figure, there is a subset of children that are infected for the majority of their first 24 months of life.

**Supplementary Figure 2. Distribution of the cumulative duration of days for which children were positive for Campylobacter spp. by qPCR and ELISA across the eight MAL-ED study sites.** The average cumulative duration of days in which children across all eight MAL-ED study sites were positive for Campylobacter spp. was 150 days (median 114 days, IQR: 28-236 days) by qPCR.

**
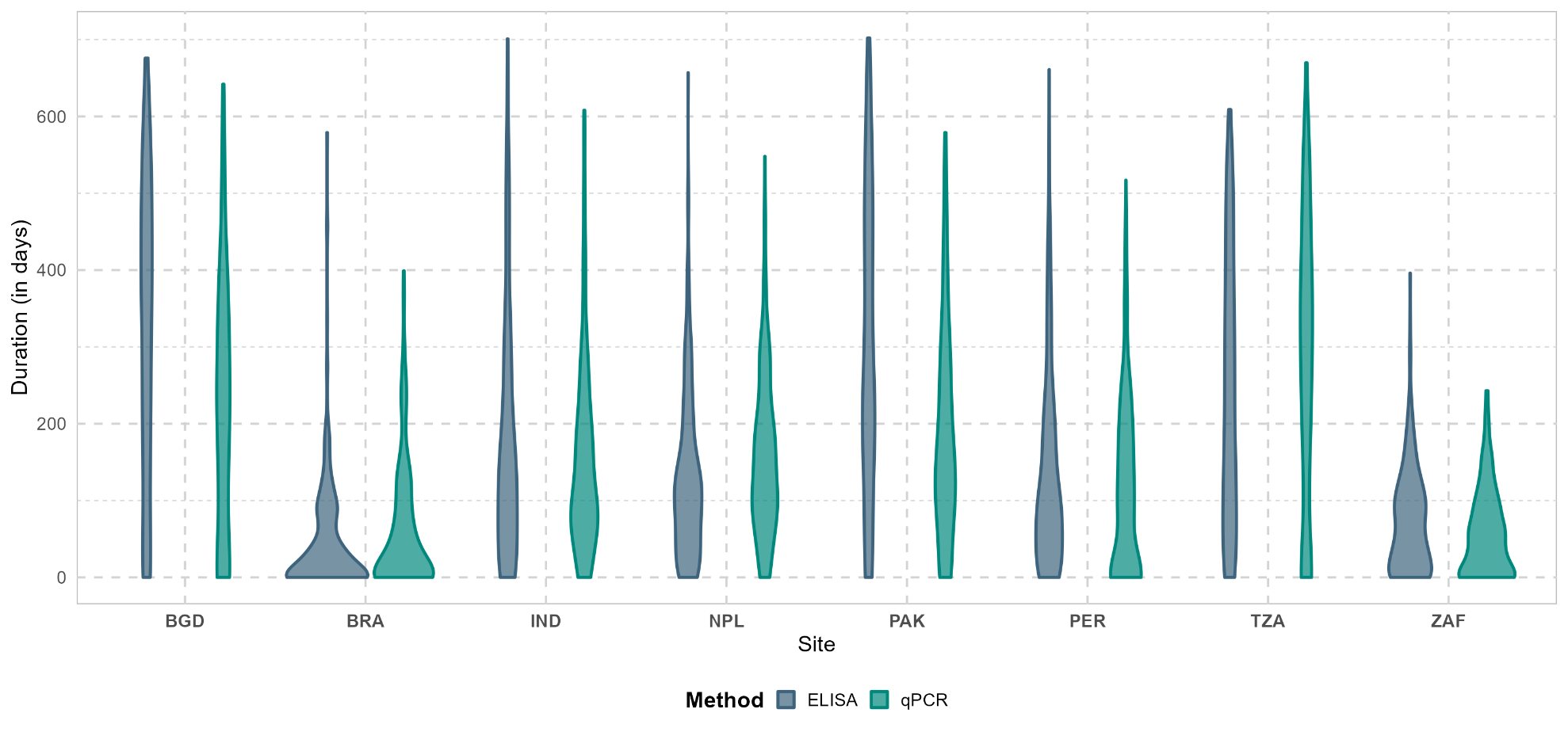
**

**Supplementary Table 1.** Unadjusted and Adjusted associations between the occurrence of persistence episodes and sociodemographic and household characteristics, across the eight MAL-ED sites, without multiple imputing missing data**.**

|  | **Unadjusted** | | | **Adjusted*** | | |
| --- | --- | --- | --- | --- | --- | --- |
|  | Odds Ratio | 95% CI | p-value | Odds Ratio | 95% CI | p-value |
| **Socio Demographic Characteristics** |  |  |  |  |  |  |
| Sex |  |  |  |  |  |  |
| *Male* | [Ref] | [Ref] | [Ref] | [Ref] | [Ref] | [Ref] |
| *Female* | 1.08 | 0.90 - 1.29 | 0.397 | 1.25 | 0.98-1.59 | 0.070 |
| Birth Weight (Kg) | 0.86 | 0.78 - 0.95 | **0.002** | 1.36 | 0.93-1.97 | 0.111 |
| Baseline LAZ | 0.95 | 0.87 - 1.03 | 0.255 | 1.15 | 0.97-1.37 | 0.111 |
| LAZ at 24 months of age | 0.64 | 0.59 - 0.70 | **<0.001** | 0.77 | 0.63-0.93 | **0.009** |
| Baseline WAZ | 0.88 | 0.81 - 0.94 | **<0.001** | 0.98 | 0.82-1.17 | 0.852 |
| WAZ at 24 months of age | 0.67 | 0.61 - 0.73 | **<0.001** | 0.92 | 0.76-1.11 | 0.415 |
| Number of diarrhea Episodes by 2nd year of life | 1.09 | 1.06-1.14 | **<0.001** | 0.85 | 0.79-0.90 | **<0.001** |
| Number of bacterial infections by 2nd year of life** | 1.25 | 1.22 - 1.29 | **<0.001** | 1.21 | 1.15-1.27 | **<0.001** |
| Number of protozoal infections by 2nd year of life | 1.35 | 1.30 - 1.40 | **<0.001** | 1.16 | 1.09-1.24 | **<0.001** |
| Number of viral infections by 2nd year of life | 1.21 | 1.18 - 1.25 | **<0.001** | 1.04 | 0.98-1.11 | 0.134 |
| Maternal Education |  |  |  |  |  |  |
| *None* | [Ref] | [Ref] | [Ref] | [Ref] | [Ref] | [Ref] |
| *Primary Complete* | 0.7 | 0.52 - 0.95 | **0.021** | 0.92 | 0.57-1.48 | 0.737 |
| *Secondary Complete* | 0.45 | 0.34 - 0.59 | **<0.001** | 0.97 | 0.59-1.57 | 0.897 |
| *Higher Education Complete* | 0.24 | 0.16 - 0.34 | **<0.001** | 1.16 | 0.60-2.26 | 0.656 |
| **WAMI Score** (Mean (SD)) | 0.05 | 0.03 - 0.08 | **<0.001** | 0.33 | 0.09-1.13 | 0.077 |
| **Household Characteristics** |  |  |  |  |  |  |
| Household Occupancy |  |  |  |  |  |  |
| *0-2 people* | [Ref] | [Ref] | [Ref] | [Ref] | [Ref] | [Ref] |
| *2-3 people* | 1.66 | 1.11 - 2.48 | **0.006** | 1.26 | 0.75-2.12 | 0.380 |
| *3-4 people* | 2.24 | 1.51 - 3.30 | **<0.001** | 1.41 | 0.83-2.39 | 0.200 |
| *>4 people* | 2.42 | 1.65 - 3.54 | **<0.001** | 1.21 | 0.71-2.07 | 0.480 |
| Water Source |  |  |  |  |  |  |
| *Unimproved* | [Ref] | [Ref] | [Ref] | [Ref] | [Ref] | [Ref] |
| *Improved* | 1.39 | 1.11 -1.74 | **0.005** | 1.30 | 0.80 - 2.10 | 0.287 |
| Sanitation |  |  |  |  |  |  |
| *Open defecation/ Unimproved* | [Ref] | [Ref] | [Ref] | [Ref] | [Ref] | [Ref] |
| *Improved* | 1.02 | 0.85 - 1.24 | 0.798 | 0.87 | 0.57-1.33 | 0.523 |
| Floor material |  |  |  |  |  |  |
| *Natural/ Rudimentary* | [Ref] | [Ref] | [Ref] | [Ref] | [Ref] | [Ref] |
| *Finished* | 0.68 | 0.57 - 0.81 | **<0.001** | 0.87 | 0.56-1.33 | 0.515 |
| Walls |  |  |  |  |  |  |
| *Natural/ Rudimentary* | [Ref] | [Ref] | [Ref] | [Ref] | [Ref] | [Ref] |
| *Finished* | 0.68 | 0.57 - 0.82 | **<0.001** | 1.02 | 0.67-1.82 | 0.9 |
| Roof |  |  |  |  |  |  |
| *Natural/ Rudimentary* | [Ref] | [Ref] | [Ref] | [Ref] | [Ref] | [Ref] |
| *Finished* | 1.35 | 1.12 - 1.62 | **0.001** | **2.48** | **1.08 - 1.99** | **0.013** |
| Poultry Ownership |  |  |  |  |  |  |
| *No* | [Ref] | [Ref] | [Ref] | [Ref] | [Ref] | [Ref] |
| *Yes* | 1.69 | 1.40 - 2.04 | **<0.001** | 1.32 | 0.96 -1.82 | 0.083 |
| Cattle Ownership |  |  |  |  |  |  |
| *No* | [Ref] | [Ref] | [Ref] | [Ref] | [Ref] | [Ref] |
| *Yes* | 2.08 | 1.66 - 2.61 | **<0.001** | 1.13 | 0.69 -1.87 | 0.625 |
| **Adjusted for all variables plus Site with fixed effects. **Excluding Campylobacter spp.* | | | | | | |

**Supplementary Table 2**. Unadjusted and Adjusted associations between the LAZ scores at 24 months of age and the occurrence of persistent Campylobacter episodes, across the eight MAL-ED sites, without multiple imputing missing data**.**

|  | **Unadjusted** | | | **Adjusted*** | | |
| --- | --- | --- | --- | --- | --- | --- |
|  | **LAZ score at 24 months** | **95% CI** | ***p-value*** | **LAZ score at 24 months** | **95% CI** | ***p-value*** |
| Persistent Campylobacter Episodes (Yes) | -0.58 | -0.69; -0.48 | **<0.001** | -0.22 | -0.30; -0.14 | **<0.001** |
| **Socio Demographic Characteristics** |  |  |  |  |  |  |
| Sex |  |  |  |  |  |  |
| *Male* | [REF] | [REF] | [REF] | [REF] | [REF] | [REF] |
| *Female* | 0.18 | 0.07; 0.28 | **0.001** | 0.12 | 0.05; 0.19 | **0.001** |
| Birth Weight (Kg) | 0.20 | 0.14; 0.26 | **<0.001** | -0.14 | -0.21; -0.08 | **<0.001** |
| Baseline LAZ | 0.64 | 0.61; 0.68 | **<0.001** | 0.47 | 0.43; 0.52 | **<0.001** |
| Baseline WAZ | 0.45 | 0.42; 0.49 | **<0.001** | 0.13 | 0.09; 0.18 | **<0.001** |
| Number of diarrhea Episodes by 2nd year of life** | -0.03 | -0.05; -0.01 | **0.002** | 0.00 | -0.00; 0.01 | 0.998 |
| Number of bacterial infections by 2nd year of life | -0.07 | -0.08; -0.06 | **<0.001** | -0.02 | -0.04; -0.01 | **0.005** |
| Number of protozoal infections by 2nd year of life | -0.12 | -0.14; -0.10 | **<0.001** | -0.03 | -0.05; -0.01 | **0.006** |
| Number of viral infections by 2nd year of life | -0.06 | -0.08; -0.05 | **<0.001** | **0.02** | **0.00; 0.04** | **0.018** |
| Maternal Education |  |  |  |  |  |  |
| *None* | [REF] | [REF] | [REF] | [REF] | [REF] | [REF] |
| *Primary Complete* | 0.33 | 0.65; 0.51 | **<0.001** | 0.08 | -0.05; 0.21 | 0.224 |
| *Secondary Complete* | 0.51 | 0.36; 0.67 | **<0.001** | 0.05 | -0.09; 0.19 | 0.476 |
| *Higher Education Complete* | 1.17 | 0.97; 1.37 | **<0.001** | 0.14 | -0.04; 0.33 | 0.129 |
| **WAMI Score** (Mean (SD)) | 2.35 | 2.13; 2.56 | **<0.001** | 0.83 | 0.48; 1.17 | **<0.001** |
| **Household Characteristics** |  |  |  |  |  |  |
| Household Occupancy |  |  |  |  |  |  |
| *0-2 people* | [REF] | [REF] | [REF] | [REF] | [REF] | [REF] |
| *2-3 people* | 0.16 | -0.05; 0.37 | 0.143 | 0.14 | -0.00; 0.28 | **0.052** |
| *3-4 people* | -0.13 | -0.33; 0.08 | 0.234 | 0.10 | -0.05; 0.24 | 0.184 |
| *>4 people* | -0.23 | -0.43; -0.03 | **0.024** | 0.05 | -0.10; 0.20 | 0.499 |
| Water Source |  |  |  |  |  |  |
| *Unimproved* | [REF] | [REF] | [REF] | [REF] | [REF] | [REF] |
| *Improved* | 0.25 | 0.11; 0.39 | **0.001** | -0.04 | -0.65; 0.52 | 0.517 |
| Sanitation |  |  |  |  |  |  |
| *Open defecation/ Unimproved* | [REF] | [REF] | [REF] | [REF] | [REF] | [REF] |
| *Improved* | 0.78 | 0.67; 0.89 | **<0.001** | -0.03 | -0.16; 0.10 | 0.625 |
| Floor material |  |  |  |  |  |  |
| *Natural/ Rudimentary* | [REF] | [REF] | [REF] | [REF] | [REF] | [REF] |
| *Finished* | 0.46 | 0.36; 0.56 | **<0.001** | 0.05 | -0.07; 0.17 | 0.427 |
| Walls |  |  |  |  |  |  |
| *Natural/ Rudimentary* | [REF] | [REF] | [REF] | [REF] | [REF] | [REF] |
| *Finished* | 0.49 | 0.39; 0.59 | **<0.001** | -0.01 | -0.13; 0.12 | 0.909 |
| Roof |  |  |  |  |  |  |
| *Natural/ Rudimentary* | [REF] | [REF] | [REF] | [REF] | [REF] | [REF] |
| *Finished* | 0.28 | 0.17; 0.39 | **<0.001** | -0.05 | -0.14; 0.04 | 0.287 |
| Poultry Ownership |  |  |  |  |  |  |
| *No* | [REF] | [REF] | [REF] | [REF] | [REF] | [REF] |
| *Yes* | -0.45 | -0.56; -0.34 | **<0.001** | 0.01 | -0.08; 0.10 | 0.828 |
| Cattle Ownership |  |  |  |  |  |  |
| *No* | [REF] | [REF] | [REF] | [REF] | [REF] | [REF] |
| *Yes* | -0.54 | -0.67; -0.40 | **<0.001** | -0.09 | -0.21; 0.03 | 0.133 |
| **Adjusted by Site. **Excluding Campylobacter spp.* |  |  |  |  |  |  |

**Supplementary Table 3.** Associations between symptomatic and asymptomatic *Campylobacter* infection of any duration and change in weight and length gain over 3-month and 9-months period, respectively, without multiple imputing missing data

|  | **Current analysis*** | **MAL-ED Peru only** | **Lee *et al*. 2013** |
| --- | --- | --- | --- |
| 9-month change in length (cm) models |  |  |  |
| Asymptomatic *Campylobacter* | 0.220 | 0.933 | -0.01 |
|  | (-0.138, 0.578) | (-0.314, 2.181) | (-0.09, 0.07) |
| Symptomatic *Campylobacter* | -0.102 | -0.018 | -0.06 |
|  | (-0.210, 0.007) | (-0.223, 0.188) | (-0.12, 0.01) |
| Non-*Campylobacter* diarrhea | -0.020 | -0.013 | -0.04 |
|  | (-0.096, 0.056) | (-0.142, 0.116) | (-0.06, -0.02) |
|  | | | |
| Asymptomatic *Campylobacter* | -18.7 | -18.8 | -65.5 |
|  | (-37.3, 0.0) | (-66.0, 28.5) | (-128.0, -3.0) |
| Symptomatic *Campylobacter* | -61.4 | -52.3 | -43.9 |
|  | (-94.8, -27.9) | (-108.8, 4.2) | (-87.6, -0.1) |
| Non-*Campylobacter* diarrhea | -23.8 | -9.9 | -22.9 |
|  | (-41.9, -5.8) | (-3.8, 1.8) | (-35.3, -10.1) |

*****Adjusted for site with fixed effects and random intercept, socio-economic status (WAMI score), prior nutritional status, sex, birthweight, episodes of diarrheal disease that were not associated *Campylobacter* in the same period, presence of non-*Campylobacter* pathogens detected over the same period.

**Supplementary Table 4.** Adjusted association between persistent Campylobacter episodes and change in weight and length gain over 3-month and 9-months period, respectively, without multiple imputing missing data**.**  Persistent episodes of increased duration are associated with greater magnitudes of linear and ponderal adverse effects on growth.

| **Length of *Campylobacter* episode*** | **Weight gain (grams/3 months)** | **Length gain (cm/9mo)** |
| --- | --- | --- |
| 1–31-day episode | *Ref* | *Ref* |
| 32–89-day episode | -19.7 | -0.122 |
|  | (-48.9, 9.4) | (-0.222, -0.023) |
| 90+ day episode (persistent) | -28.6 | -0.134 |
|  | (-6.3, 6.1) | (-0.245, -0.022) |

*****Adjusted for site with fixed effects and random intercept, socio-economic status (WAMI score), prior nutritional status, sex, birthweight, episodes of diarrheal disease that were not associated *Campylobacter* in the same period, presence of non-*Campylobacter* pathogens detected over the same period.
